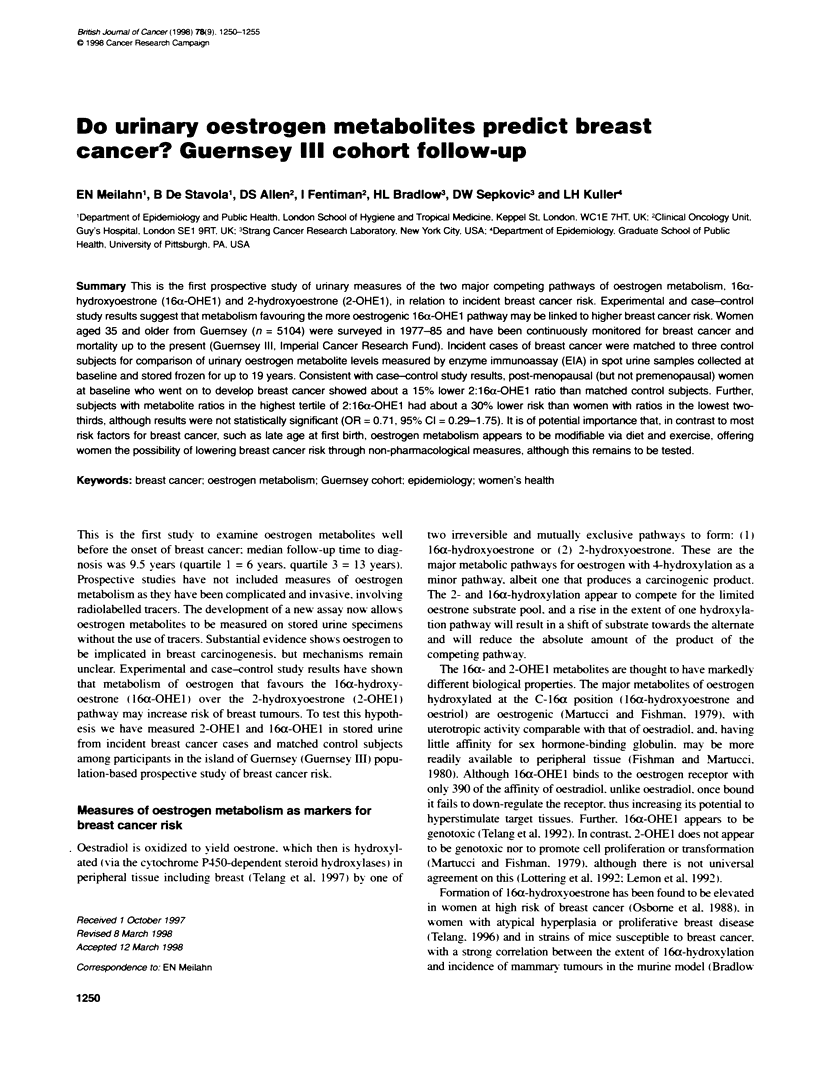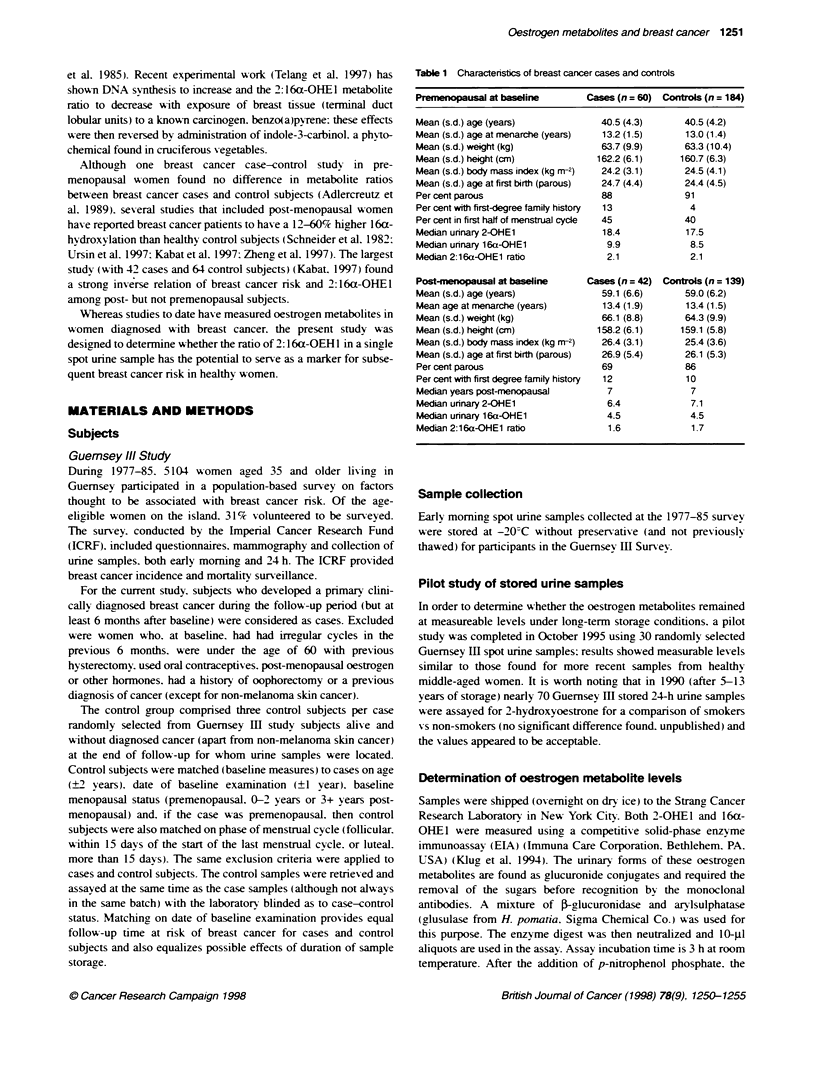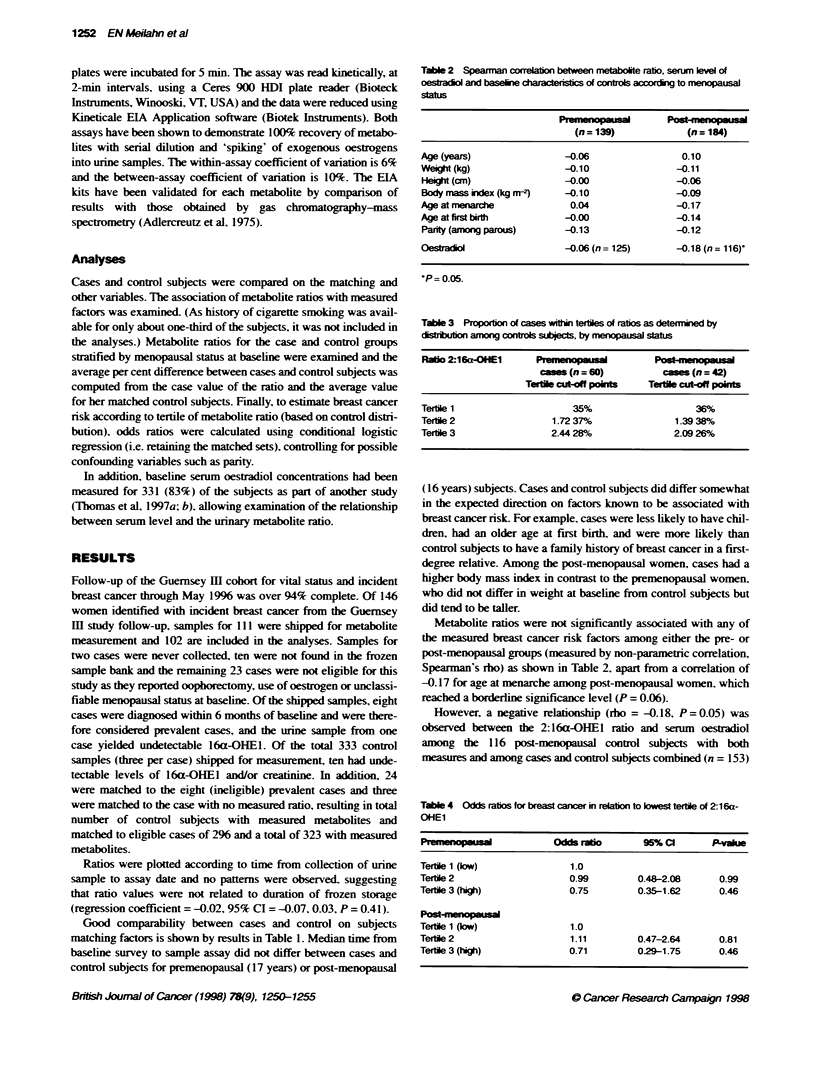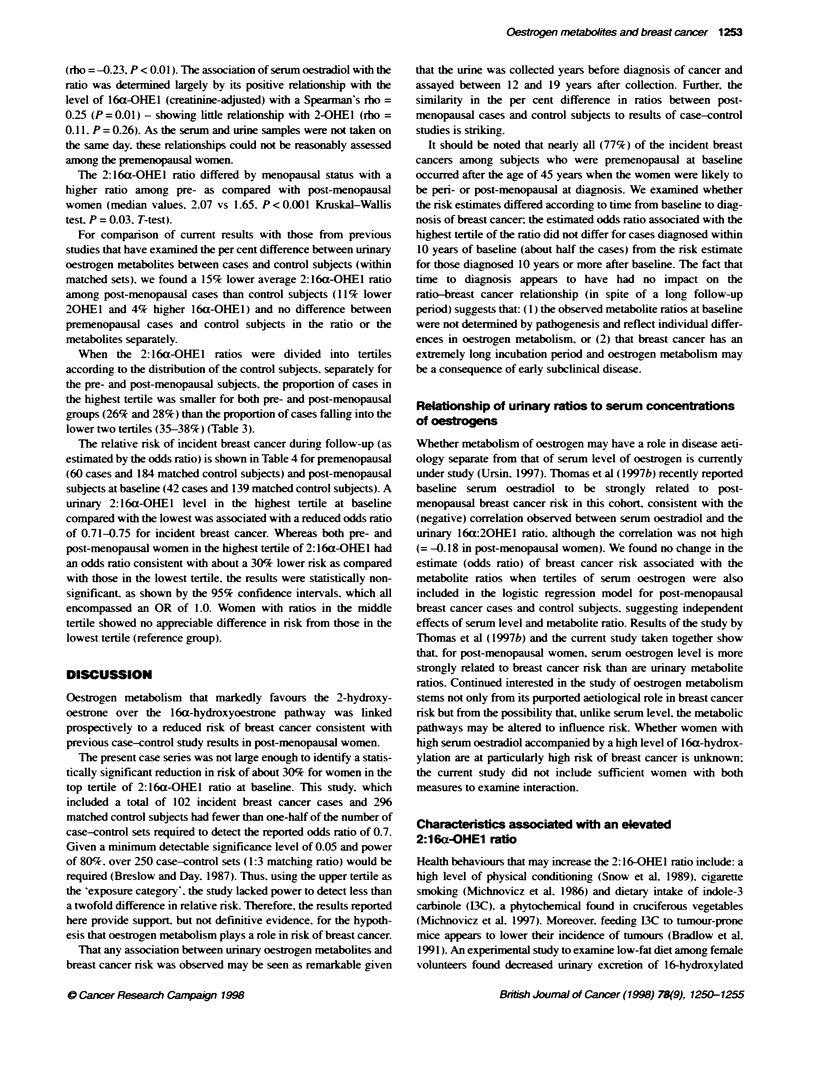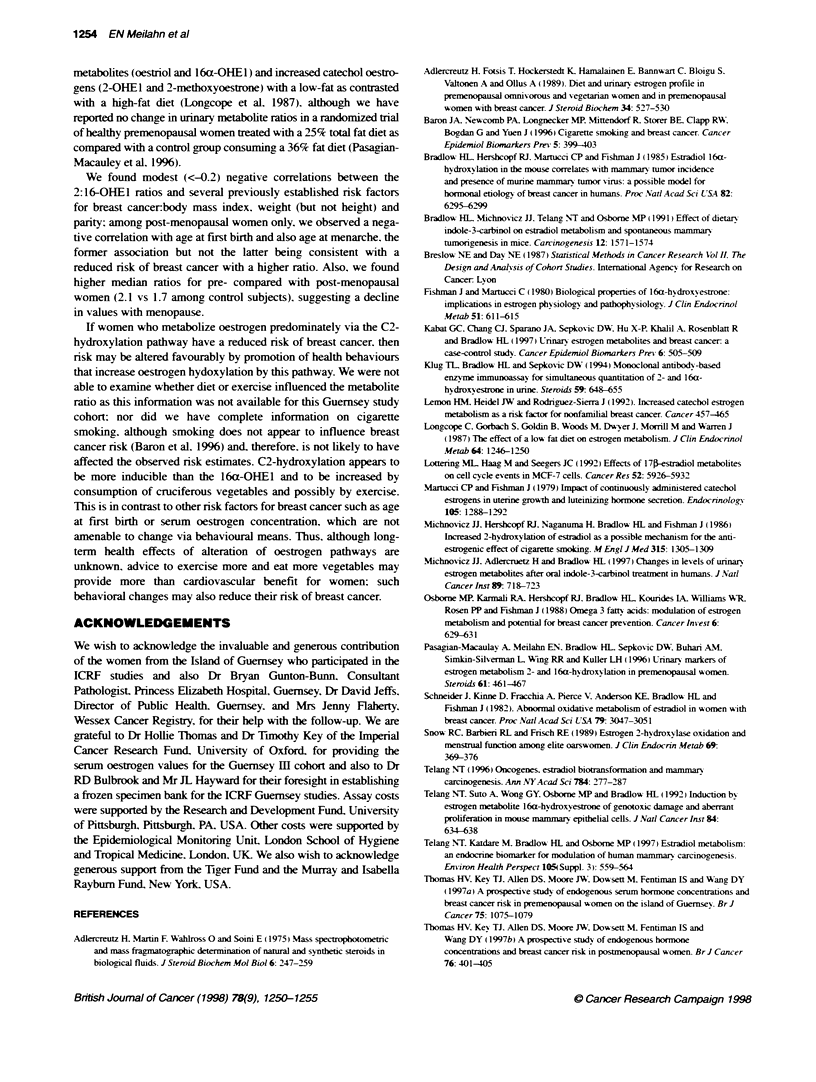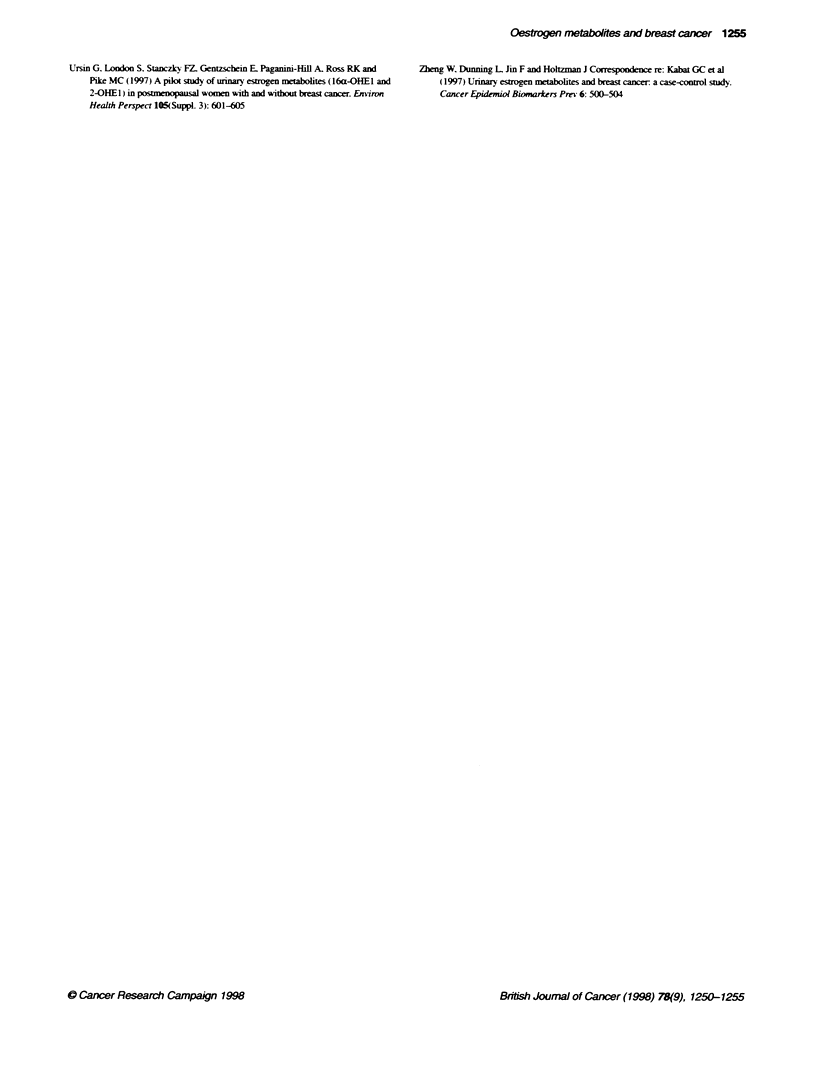# Do urinary oestrogen metabolites predict breast cancer? Guernsey III cohort follow-up.

**DOI:** 10.1038/bjc.1998.663

**Published:** 1998-11

**Authors:** E. N. Meilahn, B. De Stavola, D. S. Allen, I. Fentiman, H. L. Bradlow, D. W. Sepkovic, L. H. Kuller

**Affiliations:** Department of Epidemiology and Public Health, London School of Hygiene and Tropical Medicine, UK.

## Abstract

This is the first prospective study of urinary measures of the two major competing pathways of oestrogen metabolism, 16alpha-hydroxyoestrone (16alpha-OHE1) and 2-hydroxyoestrone (2-OHE1), in relation to incident breast cancer risk. Experimental and case-control study results suggest that metabolism favouring the more oestrogenic 16alpha-OHE1 pathway may be linked to higher breast cancer risk. Women aged 35 and older from Guernsey (n = 5104) were surveyed in 1977-85 and have been continuously monitored for breast cancer and mortality up to the present (Guernsey III, Imperial Cancer Research Fund). Incident cases of breast cancer were matched to three control subjects for comparison of urinary oestrogen metabolite levels measured by enzyme immunoassay (EIA) in spot urine samples collected at baseline and stored frozen for up to 19 years. Consistent with case-control study results, post-menopausal (but not premenopausal) women at baseline who went on to develop breast cancer showed about a 15% lower 2:16alpha-OHE1 ratio than matched control subjects. Further, subjects with metabolite ratios in the highest tertile of 2:16alpha-OHE1 had about a 30% lower risk than women with ratios in the lowest two-thirds, although results were not statistically significant (OR = 0.71, 95% CI = 0.29-1.75). It is of potential importance that, in contrast to most risk factors for breast cancer, such as late age at first birth, oestrogen metabolism appears to be modifiable via diet and exercise, offering women the possibility of lowering breast cancer risk through non-pharmacological measures, although this remains to be tested.


British Journal of Cancer (1998) 78(9), 1250–1255
© 1998 Cancer Research Campaign

### Do urinary oestrogen metabolites predict breast cancer? Guernsey III cohort follow-up

**EN Meilahn[1], B De Stavola[1], DS Allen[2], I Fentiman[2], HL Bradlow[3], DW Sepkovic[3] and LH Kuller[4]**

[1]Department of Epidemiology and Public Health, London School of Hygiene and Tropical Medicine, Keppel St, London, WC1E 7HT, UK; [2]Clinical Oncology Unit, Guy's Hospital, London SE1 9RT, UK; [3]Strang Cancer Research Laboratory, New York City, USA; [4]Department of Epidemiology, Graduate School of Public Health, University of Pittsburgh, PA, USA

**Summary** This is the first prospective study of urinary measures of the two major competing pathways of oestrogen metabolism, 16α-hydroxyoestrone (16α-OHE1) and 2-hydroxyoestrone (2-OHE1), in relation to incident breast cancer risk. Experimental and case–control study results suggest that metabolism favouring the more oestrogenic 16α-OHE1 pathway may be linked to higher breast cancer risk. Women aged 35 and older from Guernsey ($n$ = 5104) were surveyed in 1977–85 and have been continuously monitored for breast cancer and mortality up to the present (Guernsey III, Imperial Cancer Research Fund). Incident cases of breast cancer were matched to three control subjects for comparison of urinary oestrogen metabolite levels measured by enzyme immunoassay (EIA) in spot urine samples collected at baseline and stored frozen for up to 19 years. Consistent with case–control study results, post-menopausal (but not premenopausal) women at baseline who went on to develop breast cancer showed about a 15% lower 2:16α-OHE1 ratio than matched control subjects. Further, subjects with metabolite ratios in the highest tertile of 2:16α-OHE1 had about a 30% lower risk than women with ratios in the lowest two-thirds, although results were not statistically significant (OR = 0.71, 95% CI = 0.29–1.75). It is of potential importance that, in contrast to most risk factors for breast cancer, such as late age at first birth, oestrogen metabolism appears to be modifiable via diet and exercise, offering women the possibility of lowering breast cancer risk through non-pharmacological measures, although this remains to be tested.

**Keywords:** breast cancer; oestrogen metabolism; Guernsey cohort; epidemiology; women's health

This is the first study to examine oestrogen metabolites well before the onset of breast cancer; median follow-up time to diagnosis was 9.5 years (quartile 1 = 6 years, quartile 3 = 13 years). Prospective studies have not included measures of oestrogen metabolism as they have been complicated and invasive, involving radiolabelled tracers. The development of a new assay now allows oestrogen metabolites to be measured on stored urine specimens without the use of tracers. Substantial evidence shows oestrogen to be implicated in breast carcinogenesis, but mechanisms remain unclear. Experimental and case–control study results have shown that metabolism of oestrogen that favours the 16α-hydroxyoestrone (16α-OHE1) over the 2-hydroxyoestrone (2-OHE1) pathway may increase risk of breast tumours. To test this hypothesis we have measured 2-OHE1 and 16α-OHE1 in stored urine from incident breast cancer cases and matched control subjects among participants in the island of Guernsey (Guernsey III) population-based prospective study of breast cancer risk.

### Measures of oestrogen metabolism as markers for breast cancer risk

Oestradiol is oxidized to yield oestrone, which then is hydroxylated (via the cytochrome P450-dependent steroid hydroxylases) in peripheral tissue including breast (Telang et al, 1997) by one of two irreversible and mutually exclusive pathways to form: (1) 16α-hydroxyoestrone or (2) 2-hydroxyoestrone. These are the major metabolic pathways for oestrogen with 4-hydroxylation as a minor pathway, albeit one that produces a carcinogenic product. The 2- and 16α-hydroxylation appear to compete for the limited oestrone substrate pool, and a rise in the extent of one hydroxylation pathway will result in a shift of substrate towards the alternate and will reduce the absolute amount of the product of the competing pathway.

The 16α- and 2-OHE1 metabolites are thought to have markedly different biological properties. The major metabolites of oestrogen hydroxylated at the C-16α position (16α-hydroxyoestrone and oestriol) are oestrogenic (Martucci and Fishman, 1979), with uterotropic activity comparable with that of oestradiol, and, having little affinity for sex hormone-binding globulin, may be more readily available to peripheral tissue (Fishman and Martucci, 1980). Although 16α-OHE1 binds to the oestrogen receptor with only 390 of the affinity of oestradiol, unlike oestradiol, once bound it fails to down-regulate the receptor, thus increasing its potential to hyperstimulate target tissues. Further, 16α-OHE1 appears to be genotoxic (Telang et al, 1992). In contrast, 2-OHE1 does not appear to be genotoxic nor to promote cell proliferation or transformation (Martucci and Fishman, 1979), although there is not universal agreement on this (Lottering et al, 1992; Lemon et al, 1992).

Formation of 16α-hydroxyoestrone has been found to be elevated in women at high risk of breast cancer (Osborne et al, 1988), in women with atypical hyperplasia or proliferative breast disease (Telang, 1996) and in strains of mice susceptible to breast cancer, with a strong correlation between the extent of 16α-hydroxylation and incidence of mammary tumours in the murine model (Bradlow

*Received 1 October 1997*
*Revised 8 March 1998*
*Accepted 12 March 1998*

*Correspondence to:* EN Meilahn et al. 1985). Recent experimental work (Telang et al. 1997) has shown DNA synthesis to increase and the 2:16α-OHE1 metabolite ratio to decrease with exposure of breast tissue (terminal duct lobular units) to a known carcinogen, benzo(a)pyrene; these effects were then reversed by administration of indole-3-carbinol, a phytochemical found in cruciferous vegetables.

Although one breast cancer case–control study in premenopausal women found no difference in metabolite ratios between breast cancer cases and control subjects (Adlercreutz et al. 1989), several studies that included post-menopausal women have reported breast cancer patients to have a 12–60% higher 16α-hydroxylation than healthy control subjects (Schneider et al. 1982; Ursin et al. 1997; Kabat et al. 1997; Zheng et al. 1997). The largest study (with 42 cases and 64 control subjects) (Kabat, 1997) found a strong inverse relation of breast cancer risk and 2:16α-OHE1 among post- but not premenopausal subjects.

Whereas studies to date have measured oestrogen metabolites in women diagnosed with breast cancer, the present study was designed to determine whether the ratio of 2:16α-OEH1 in a single spot urine sample has the potential to serve as a marker for subsequent breast cancer risk in healthy women.

### MATERIALS AND METHODS

### Subjects

#### *Guernsey III Study*

During 1977–85, 5104 women aged 35 and older living in Guernsey participated in a population-based survey on factors thought to be associated with breast cancer risk. Of the age-eligible women on the island, 31% volunteered to be surveyed. The survey, conducted by the Imperial Cancer Research Fund (ICRF), included questionnaires, mammography and collection of urine samples, both early morning and 24 h. The ICRF provided breast cancer incidence and mortality surveillance.

For the current study, subjects who developed a primary clinically diagnosed breast cancer during the follow-up period (but at least 6 months after baseline) were considered as cases. Excluded were women who, at baseline, had had irregular cycles in the previous 6 months, were under the age of 60 with previous hysterectomy, used oral contraceptives, post-menopausal oestrogen or other hormones, had a history of oophorectomy or a previous diagnosis of cancer (except for non-melanoma skin cancer).

The control group comprised three control subjects per case randomly selected from Guernsey III study subjects alive and without diagnosed cancer (apart from non-melanoma skin cancer) at the end of follow-up for whom urine samples were located. Control subjects were matched (baseline measures) to cases on age (±2 years), date of baseline examination (±1 year), baseline menopausal status (premenopausal, 0–2 years or 3+ years post-menopausal) and, if the case was premenopausal, then control subjects were also matched on phase of menstrual cycle (follicular, within 15 days of the start of the last menstrual cycle, or luteal, more than 15 days). The same exclusion criteria were applied to cases and control subjects. The control samples were retrieved and assayed at the same time as the case samples (although not always in the same batch) with the laboratory blinded as to case–control status. Matching on date of baseline examination provides equal follow-up time at risk of breast cancer for cases and control subjects and also equalizes possible effects of duration of sample storage.

**Table 1** Characteristics of breast cancer cases and controls

| **Premenopausal at baseline** | **Cases ($n$ = 60)** | **Controls ($n$ = 184)** |
|---|---|---|
| Mean (s.d.) age (years) | 40.5 (4.3) | 40.5 (4.2) |
| Mean (s.d.) age at menarche (years) | 13.2 (1.5) | 13.0 (1.4) |
| Mean (s.d.) weight (kg) | 63.7 (9.9) | 63.3 (10.4) |
| Mean (s.d.) height (cm) | 162.2 (6.1) | 160.7 (6.3) |
| Mean (s.d.) body mass index (kg m$^{-2}$) | 24.2 (3.1) | 24.5 (4.1) |
| Mean (s.d.) age at first birth (parous) | 24.7 (4.4) | 24.4 (4.5) |
| Per cent parous | 88 | 91 |
| Per cent with first-degree family history | 13 | 4 |
| Per cent in first half of menstrual cycle | 45 | 40 |
| Median urinary 2-OHE1 | 18.4 | 17.5 |
| Median urinary 16α-OHE1 | 9.9 | 8.5 |
| Median 2:16α-OHE1 ratio | 2.1 | 2.1 |
| **Post-menopausal at baseline** | **Cases ($n$ = 42)** | **Controls ($n$ = 139)** |
| Mean (s.d.) age (years) | 59.1 (6.6) | 59.0 (6.2) |
| Mean age at menarche (years) | 13.4 (1.9) | 13.4 (1.5) |
| Mean (s.d.) weight (kg) | 66.1 (8.8) | 64.3 (9.9) |
| Mean (s.d.) height (cm) | 158.2 (6.1) | 159.1 (5.8) |
| Mean (s.d.) body mass index (kg m$^{-2}$) | 26.4 (3.1) | 25.4 (3.6) |
| Mean (s.d.) age at first birth (parous) | 26.9 (5.4) | 26.1 (5.3) |
| Per cent parous | 69 | 86 |
| Per cent with first degree family history | 12 | 10 |
| Median years post-menopausal | 7 | 7 |
| Median urinary 2-OHE1 | 6.4 | 7.1 |
| Median urinary 16α-OHE1 | 4.5 | 4.5 |
| Median 2:16α-OHE1 ratio | 1.6 | 1.7 |

### Sample collection

Early morning spot urine samples collected at the 1977–85 survey were stored at –20°C without preservative (and not previously thawed) for participants in the Guernsey III Survey.

### Pilot study of stored urine samples

In order to determine whether the oestrogen metabolites remained at measureable levels under long-term storage conditions, a pilot study was completed in October 1995 using 30 randomly selected Guernsey III spot urine samples; results showed measurable levels similar to those found for more recent samples from healthy middle-aged women. It is worth noting that in 1990 (after 5–13 years of storage) nearly 70 Guernsey III stored 24-h urine samples were assayed for 2-hydroxyoestrone for a comparison of smokers vs non-smokers (no significant difference found, unpublished) and the values appeared to be acceptable.

### Determination of oestrogen metabolite levels

Samples were shipped (overnight on dry ice) to the Strang Cancer Research Laboratory in New York City. Both 2-OHE1 and 16α-OHE1 were measured using a competitive solid-phase enzyme immunoassay (EIA) (Immuna Care Corporation, Bethlehem, PA, USA) (Klug et al. 1994). The urinary forms of these oestrogen metabolites are found as glucuronide conjugates and required the removal of the sugars before recognition by the monoclonal antibodies. A mixture of β-glucuronidase and arylsulphatase (glusulase from *H. pomatia*, Sigma Chemical Co.) was used for this purpose. The enzyme digest was then neutralized and 10-µl aliquots are used in the assay. Assay incubation time is 3 h at room temperature. After the addition of *p*-nitrophenol phosphate, the

© Cancer Research Campaign 1998

plates were incubated for 5 min. The assay was read kinetically, at 2-min intervals, using a Ceres 900 HDI plate reader (Bioteck Instruments, Winooski, VT, USA) and the data were reduced using Kineticale EIA Application software (Biotek Instruments). Both assays have been shown to demonstrate 100% recovery of metabolites with serial dilution and 'spiking' of exogenous oestrogens into urine samples. The within-assay coefficient of variation is 6% and the between-assay coefficient of variation is 10%. The EIA kits have been validated for each metabolite by comparison of results with those obtained by gas chromatography–mass spectrometry (Adlercreutz et al, 1975).

### Analyses

Cases and control subjects were compared on the matching and other variables. The association of metabolite ratios with measured factors was examined. (As history of cigarette smoking was available for only about one-third of the subjects, it was not included in the analyses.) Metabolite ratios for the case and control groups stratified by menopausal status at baseline were examined and the average per cent difference between cases and control subjects was computed from the case value of the ratio and the average value for her matched control subjects. Finally, to estimate breast cancer risk according to tertile of metabolite ratio (based on control distribution), odds ratios were calculated using conditional logistic regression (i.e. retaining the matched sets), controlling for possible confounding variables such as parity.

In addition, baseline serum oestradiol concentrations had been measured for 331 (83%) of the subjects as part of another study (Thomas et al, 1997*a*; *b*), allowing examination of the relationship between serum level and the urinary metabolite ratio.

### RESULTS

Follow-up of the Guernsey III cohort for vital status and incident breast cancer through May 1996 was over 94% complete. Of 146 women identified with incident breast cancer from the Guernsey III study follow-up, samples for 111 were shipped for metabolite measurement and 102 are included in the analyses. Samples for two cases were never collected, ten were not found in the frozen sample bank and the remaining 23 cases were not eligible for this study as they reported oophorectomy, use of oestrogen or unclassifiable menopausal status at baseline. Of the shipped samples, eight cases were diagnosed within 6 months of baseline and were therefore considered prevalent cases, and the urine sample from one case yielded undetectable 16α-OHE1. Of the total 333 control samples (three per case) shipped for measurement, ten had undetectable levels of 16α-OHE1 and/or creatinine. In addition, 24 were matched to the eight (ineligible) prevalent cases and three were matched to the case with no measured ratio, resulting in total number of control subjects with measured metabolites and matched to eligible cases of 296 and a total of 323 with measured metabolites.

Ratios were plotted according to time from collection of urine sample to assay date and no patterns were observed, suggesting that ratio values were not related to duration of frozen storage (regression coefficient = –0.02, 95% CI = –0.07, 0.03, $P = 0.41$).

Good comparability between cases and control on subjects matching factors is shown by results in Table 1. Median time from baseline survey to sample assay did not differ between cases and control subjects for premenopausal (17 years) or post-menopausal (16 years) subjects. Cases and control subjects did differ somewhat in the expected direction on factors known to be associated with breast cancer risk. For example, cases were less likely to have children, had an older age at first birth, and were more likely than control subjects to have a family history of breast cancer in a first-degree relative. Among the post-menopausal women, cases had a higher body mass index in contrast to the premenopausal women, who did not differ in weight at baseline from control subjects but did tend to be taller.

Metabolite ratios were not significantly associated with any of the measured breast cancer risk factors among either the pre- or post-menopausal groups (measured by non-parametric correlation, Spearman's rho) as shown in Table 2, apart from a correlation of –0.17 for age at menarche among post-menopausal women, which reached a borderline significance level ($P = 0.06$).

However, a negative relationship (rho = –0.18, $P = 0.05$) was observed between the 2:16α-OHE1 ratio and serum oestradiol among the 116 post-menopausal control subjects with both measures and among cases and control subjects combined ($n = 153$)

**Table 2** Spearman correlation between metabolite ratio, serum level of oestradiol and baseline characteristics of controls according to menopausal status

| | Premenopausal ($n = 139$) | Post-menopausal ($n = 184$) |
|---|---|---|
| Age (years) | –0.06 | 0.10 |
| Weight (kg) | –0.10 | –0.11 |
| Height (cm) | –0.00 | –0.06 |
| Body mass index (kg $m^{-2}$) | –0.10 | –0.09 |
| Age at menarche | 0.04 | –0.17 |
| Age at first birth | –0.00 | –0.14 |
| Parity (among parous) | –0.13 | –0.12 |
| Oestradiol | –0.06 ($n = 125$) | –0.18 ($n = 116$)* |

*$P = 0.05$.

**Table 3** Proportion of cases within tertiles of ratios as determined by distribution among controls subjects, by menopausal status

| Ratio 2:16α-OHE1 | Premenopausal cases ($n = 60$) Tertile cut-off points | Post-menopausal cases ($n = 42$) Tertile cut-off points |
|---|---|---|
| Tertile 1 | 35% | 36% |
| Tertile 2 | 1.72 37% | 1.39 38% |
| Tertile 3 | 2.44 28% | 2.09 26% |

**Table 4** Odds ratios for breast cancer in relation to lowest tertile of 2:16α-OHE1

| Premenopausal | Odds ratio | 95% CI | *P*-value |
|---|---|---|---|
| Tertile 1 (low) | 1.0 | | |
| Tertile 2 | 0.99 | 0.48–2.08 | 0.99 |
| Tertile 3 (high) | 0.75 | 0.35–1.62 | 0.46 |
| **Post-menopausal** | | | |
| Tertile 1 (low) | 1.0 | | |
| Tertile 2 | 1.11 | 0.47–2.64 | 0.81 |
| Tertile 3 (high) | 0.71 | 0.29–1.75 | 0.46 |

© Cancer Research Campaign 1998

(rho = –0.23, $P < 0.01$). The association of serum oestradiol with the ratio was determined largely by its positive relationship with the level of 16α-OHE1 (creatinine-adjusted) with a Spearman's rho = 0.25 ($P = 0.01$) – showing little relationship with 2-OHE1 (rho = 0.11, $P = 0.26$). As the serum and urine samples were not taken on the same day, these relationships could not be reasonably assessed among the premenopausal women.

The 2:16α-OHE1 ratio differed by menopausal status with a higher ratio among pre- as compared with post-menopausal women (median values, 2.07 vs 1.65, $P < 0.001$ Kruskal–Wallis test, $P = 0.03$, *T*-test).

For comparison of current results with those from previous studies that have examined the per cent difference between urinary oestrogen metabolites between cases and control subjects (within matched sets), we found a 15% lower average 2:16α-OHE1 ratio among post-menopausal cases than control subjects (11% lower 2OHE1 and 4% higher 16α-OHE1) and no difference between premenopausal cases and control subjects in the ratio or the metabolites separately.

When the 2:16α-OHE1 ratios were divided into tertiles according to the distribution of the control subjects, separately for the pre- and post-menopausal subjects, the proportion of cases in the highest tertile was smaller for both pre- and post-menopausal groups (26% and 28%) than the proportion of cases falling into the lower two tertiles (35–38%) (Table 3).

The relative risk of incident breast cancer during follow-up (as estimated by the odds ratio) is shown in Table 4 for premenopausal (60 cases and 184 matched control subjects) and post-menopausal subjects at baseline (42 cases and 139 matched control subjects). A urinary 2:16α-OHE1 level in the highest tertile at baseline compared with the lowest was associated with a reduced odds ratio of 0.71–0.75 for incident breast cancer. Whereas both pre- and post-menopausal women in the highest tertile of 2:16α-OHE1 had an odds ratio consistent with about a 30% lower risk as compared with those in the lowest tertile, the results were statistically non-significant, as shown by the 95% confidence intervals, which all encompassed an OR of 1.0. Women with ratios in the middle tertile showed no appreciable difference in risk from those in the lowest tertile (reference group).

### DISCUSSION

Oestrogen metabolism that markedly favours the 2-hydroxyoestrone over the 16α-hydroxyoestrone pathway was linked prospectively to a reduced risk of breast cancer consistent with previous case–control study results in post-menopausal women.

The present case series was not large enough to identify a statistically significant reduction in risk of about 30% for women in the top tertile of 2:16α-OHE1 ratio at baseline. This study, which included a total of 102 incident breast cancer cases and 296 matched control subjects had fewer than one-half of the number of case–control sets required to detect the reported odds ratio of 0.7. Given a minimum detectable significance level of 0.05 and power of 80%, over 250 case–control sets (1:3 matching ratio) would be required (Breslow and Day, 1987). Thus, using the upper tertile as the 'exposure category', the study lacked power to detect less than a twofold difference in relative risk. Therefore, the results reported here provide support, but not definitive evidence, for the hypothesis that oestrogen metabolism plays a role in risk of breast cancer.

That any association between urinary oestrogen metabolites and breast cancer risk was observed may be seen as remarkable given that the urine was collected years before diagnosis of cancer and assayed between 12 and 19 years after collection. Further, the similarity in the per cent difference in ratios between post-menopausal cases and control subjects to results of case–control studies is striking.

It should be noted that nearly all (77%) of the incident breast cancers among subjects who were premenopausal at baseline occurred after the age of 45 years when the women were likely to be peri- or post-menopausal at diagnosis. We examined whether the risk estimates differed according to time from baseline to diagnosis of breast cancer; the estimated odds ratio associated with the highest tertile of the ratio did not differ for cases diagnosed within 10 years of baseline (about half the cases) from the risk estimate for those diagnosed 10 years or more after baseline. The fact that time to diagnosis appears to have had no impact on the ratio–breast cancer relationship (in spite of a long follow-up period) suggests that: (1) the observed metabolite ratios at baseline were not determined by pathogenesis and reflect individual differences in oestrogen metabolism, or (2) that breast cancer has an extremely long incubation period and oestrogen metabolism may be a consequence of early subclinical disease.

### Relationship of urinary ratios to serum concentrations of oestrogens

Whether metabolism of oestrogen may have a role in disease aetiology separate from that of serum level of oestrogen is currently under study (Ursin, 1997). Thomas et al (1997*b*) recently reported baseline serum oestradiol to be strongly related to post-menopausal breast cancer risk in this cohort, consistent with the (negative) correlation observed between serum oestradiol and the urinary 16α:2OHE1 ratio, although the correlation was not high (= –0.18 in post-menopausal women). We found no change in the estimate (odds ratio) of breast cancer risk associated with the metabolite ratios when tertiles of serum oestrogen were also included in the logistic regression model for post-menopausal breast cancer cases and control subjects, suggesting independent effects of serum level and metabolite ratio. Results of the study by Thomas et al (1997*b*) and the current study taken together show that, for post-menopausal women, serum oestrogen level is more strongly related to breast cancer risk than are urinary metabolite ratios. Continued interested in the study of oestrogen metabolism stems not only from its purported aetiological role in breast cancer risk but from the possibility that, unlike serum level, the metabolic pathways may be altered to influence risk. Whether women with high serum oestradiol accompanied by a high level of 16α-hydroxylation are at particularly high risk of breast cancer is unknown; the current study did not include sufficient women with both measures to examine interaction.

### Characteristics associated with an elevated 2:16α-OHE1 ratio

Health behaviours that may increase the 2:16-OHE1 ratio include: a high level of physical conditioning (Snow et al, 1989), cigarette smoking (Michnovicz et al, 1986) and dietary intake of indole-3 carbinole (I3C), a phytochemical found in cruciferous vegetables (Michnovicz et al, 1997). Moreover, feeding I3C to tumour-prone mice appears to lower their incidence of tumours (Bradlow et al, 1991). An experimental study to examine low-fat diet among female volunteers found decreased urinary excretion of 16-hydroxylated

© Cancer Research Campaign 1998

metabolites (oestriol and 16α-OHE1) and increased catechol oestrogens (2-OHE1 and 2-methoxyoestrone) with a low-fat as contrasted with a high-fat diet (Longcope et al. 1987), although we have reported no change in urinary metabolite ratios in a randomized trial of healthy premenopausal women treated with a 25% total fat diet as compared with a control group consuming a 36% fat diet (Pasagian-Macauley et al. 1996).

We found modest (<–0.2) negative correlations between the 2:16-OHE1 ratios and several previously established risk factors for breast cancer:body mass index, weight (but not height) and parity; among post-menopausal women only, we observed a negative correlation with age at first birth and also age at menarche, the former association but not the latter being consistent with a reduced risk of breast cancer with a higher ratio. Also, we found higher median ratios for pre- compared with post-menopausal women (2.1 vs 1.7 among control subjects), suggesting a decline in values with menopause.

If women who metabolize oestrogen predominately via the C2-hydroxylation pathway have a reduced risk of breast cancer, then risk may be altered favourably by promotion of health behaviours that increase oestrogen hydoxylation by this pathway. We were not able to examine whether diet or exercise influenced the metabolite ratio as this information was not available for this Guernsey study cohort; nor did we have complete information on cigarette smoking, although smoking does not appear to influence breast cancer risk (Baron et al. 1996) and, therefore, is not likely to have affected the observed risk estimates. C2-hydroxylation appears to be more inducible than the 16α-OHE1 and to be increased by consumption of cruciferous vegetables and possibly by exercise. This is in contrast to other risk factors for breast cancer such as age at first birth or serum oestrogen concentration, which are not amenable to change via behavioural means. Thus, although long-term health effects of alteration of oestrogen pathways are unknown, advice to exercise more and eat more vegetables may provide more than cardiovascular benefit for women; such behavioral changes may also reduce their risk of breast cancer.

### ACKNOWLEDGEMENTS

We wish to acknowledge the invaluable and generous contribution of the women from the Island of Guernsey who participated in the ICRF studies and also Dr Bryan Gunton-Bunn, Consultant Pathologist, Princess Elizabeth Hospital, Guernsey, Dr David Jeffs, Director of Public Health, Guernsey, and Mrs Jenny Flaherty, Wessex Cancer Registry, for their help with the follow-up. We are grateful to Dr Hollie Thomas and Dr Timothy Key of the Imperial Cancer Research Fund, University of Oxford, for providing the serum oestrogen values for the Guernsey III cohort and also to Dr RD Bulbrook and Mr JL Hayward for their foresight in establishing a frozen specimen bank for the ICRF Guernsey studies. Assay costs were supported by the Research and Development Fund, University of Pittsburgh, Pittsburgh, PA, USA. Other costs were supported by the Epidemiological Monitoring Unit, London School of Hygiene and Tropical Medicine, London, UK. We also wish to acknowledge generous support from the Tiger Fund and the Murray and Isabella Rayburn Fund, New York, USA.

### REFERENCES

Adlercreutz H, Martin F, Wahlross O and Soini E (1975) Mass spectrophotometric and mass fragmatographic determination of natural and synthetic steroids in biological fluids. *J Steroid Biochem Mol Biol* **6**: 247–259

Adlercreutz H, Fotsis T, Hockerstedt K, Hamalainen E, Bannwart C, Bloigu S, Valtonen A and Ollus A (1989). Diet and urinary estrogen profile in premenopausal omnivorous and vegetarian women and in premenopausal women with breast cancer. *J Steroid Biochem* **34**: 527–530

Baron JA, Newcomb PA, Longnecker MP, Mittendorf R, Storer BE, Clapp RW, Bogdan G and Yuen J (1996) Cigarette smoking and breast cancer. *Cancer Epidemiol Biomarkers Prev* **5**: 399–403

Bradlow HL, Hershcopf RJ, Martucci CP and Fishman J (1985) Estradiol 16α-hydroxylation in the mouse correlates with mammary tumor incidence and presence of murine mammary tumor virus: a possible model for hormonal etiology of breast cancer in humans. *Proc Natl Acad Sci USA* **82**: 6295–6299

Bradlow HL, Michnovicz JJ, Telang NT and Osborne MP (1991) Effect of dietary indole-3-carbinol on estradiol metabolism and spontaneous mammary tumorigenesis in mice. *Carcinogenesis* **12**: 1571–1574

Breslow NE and Day NE (1987) *Statistical Methods in Cancer Research Vol II. The Design and Analysis of Cohort Studies*. International Agency for Research on Cancer: Lyon

Fishman J and Martucci C (1980) Biological properties of 16α-hydroxyestrone: implications in estrogen physiology and pathophysiology. *J Clin Endocrinol Metab* **51**: 611–615

Kabat GC, Chang CJ, Sparano JA, Sepkovic DW, Hu X-P, Khalil A, Rosenblatt R and Bradlow HL (1997) Urinary estrogen metabolites and breast cancer: a case-control study. *Cancer Epidemiol Biomarkers Prev* **6**: 505–509

Klug TL, Bradlow HL and Sepkovic DW (1994) Monoclonal antibody-based enzyme immunoassay for simultaneous quantitation of 2- and 16α-hydroxyestrone in urine. *Steroids* **59**: 648–655

Lemon HM, Heidel JW and Rodriguez-Sierra J (1992). Increased catechol estrogen metabolism as a risk factor for nonfamilial breast cancer. *Cancer* 457–465

Longcope C, Gorbach S, Goldin B, Woods M, Dwyer J, Morrill M and Warren J (1987) The effect of a low fat diet on estrogen metabolism. *J Clin Endocrinol Metab* **64**: 1246–1250

Lottering ML, Haag M and Seegers JC (1992) Effects of 17β-estradiol metabolites on cell cycle events in MCF-7 cells. *Cancer Res* **52**: 5926–5932

Martucci CP and Fishman J (1979) Impact of continuously administered catechol estrogens in uterine growth and luteinizing hormone secretion. *Endocrinology* **105**: 1288–1292

Michnovicz JJ, Hershcopf RJ, Naganuma H, Bradlow HL and Fishman J (1986) Increased 2-hydroxylation of estradiol as a possible mechanism for the anti-estrogenic effect of cigarette smoking. *M Engl J Med* **315**: 1305–1309

Michnovicz JJ, Adlercruetz H and Bradlow HL (1997) Changes in levels of urinary estrogen metabolites after oral indole-3-carbinol treatment in humans. *J Natl Cancer Inst* **89**: 718–723

Osborne MP, Karmali RA, Hershcopf RJ, Bradlow HL, Kourides IA, Williams WR, Rosen PP and Fishman J (1988) Omega 3 fatty acids: modulation of estrogen metabolism and potential for breast cancer prevention. *Cancer Invest* **6**: 629–631

Pasagian-Macaulay A, Meilahn EN, Bradlow HL, Sepkovic DW, Buhari AM, Simkin-Silverman L, Wing RR and Kuller LH (1996) Urinary markers of estrogen metabolism 2- and 16α-hydroxylation in premenopausal women. *Steroids* **61**: 461–467

Schneider J, Kinne D, Fracchia A, Pierce V, Anderson KE, Bradlow HL and Fishman J (1982). Abnormal oxidative metabolism of estradiol in women with breast cancer. *Proc Natl Acad Sci USA* **79**: 3047–3051

Snow RC, Barbieri RL and Frisch RE (1989) Estrogen 2-hydroxylase oxidation and menstrual function among elite oarswomen. *J Clin Endocrin Metab* **69**: 369–376

Telang NT (1996) Oncogenes, estradiol biotransformation and mammary carcinogenesis. *Ann NY Acad Sci* **784**: 277–287

Telang NT, Suto A, Wong GY, Osborne MP and Bradlow HL (1992) Induction by estrogen metabolite 16α-hydroxyestrone of genotoxic damage and aberrant proliferation in mouse mammary epithelial cells. *J Natl Cancer Inst* **84**: 634–638

Telang NT, Katdare M, Bradlow HL and Osborne MP (1997) Estradiol metabolism: an endocrine biomarker for modulation of human mammary carcinogenesis. *Environ Health Perspect* **105**(Suppl. 3): 559–564

Thomas HV, Key TJ, Allen DS, Moore JW, Dowsett M, Fentiman IS and Wang DY (1997*a*) A prospective study of endogenous serum hormone concentrations and breast cancer risk in premenopausal women on the island of Guernsey. *Br J Cancer* **75**: 1075–1079

Thomas HV, Key TJ, Allen DS, Moore JW, Dowsett M, Fentiman IS and Wang DY (1997*b*) A prospective study of endogenous hormone concentrations and breast cancer risk in postmenopausal women. *Br J Cancer* **76**: 401–405

© Cancer Research Campaign 1998

Ursin G, London S, Stanczky FZ, Gentzschein E, Paganini-Hill A, Ross RK and Pike MC (1997) A pilot study of urinary estrogen metabolites (16α-OHE1 and 2-OHE1) in postmenopausal women with and without breast cancer. *Environ Health Perspect* **105**(Suppl. 3): 601–605

Zheng W, Dunning L, Jin F and Holtzman J Correspondence re: Kabat GC et al (1997) Urinary estrogen metabolites and breast cancer: a case-control study. *Cancer Epidemiol Biomarkers Prev* **6**: 500–504

© Cancer Research Campaign 1998